# Differences in immune response to anesthetics used for day surgery versus hospitalization surgery for breast cancer patients

**DOI:** 10.1186/s40169-017-0163-4

**Published:** 2017-09-14

**Authors:** Ryungsa Kim, Ami Kawai, Megumi Wakisaka, Yuri Funaoka, Shoichiro Ohtani, Mitsuya Ito, Takayuki Kadoya, Morihito Okada

**Affiliations:** 1 Department of Breast Surgery, Hiroshima Mark Clinic, 1-4-3F, 2-chome, Ohte-machi, Naka-ku, Hiroshima, 730-0051 Japan; 2 Department of Breast Surgery, Hiroshima City Hospital, 7-33 Moto-machi, Naka-ku, Hiroshima, 730-8518 Japan; 3 Department of Surgical Oncology, Research Institute for Radiation Biology and Medicine, Hiroshima University, 1-2-3-Kasumi, Minami-ku, Hiroshima, 734-0037 Japan

**Keywords:** Immune response, Anesthetic, Day surgery, Hospitalization surgery, Breast cancer

## Abstract

**Background:**

Surgery/anesthetic technique-stimulated immunosuppression may be associated with outcome for cancer patients. Here, the immune responses of patients undergoing day surgery versus hospitalization surgery for breast cancer were compared in a prospective study.

**Methods:**

Between February 2012 and August 2014, 21 breast cancer patients underwent day surgery and 16 breast cancer patients underwent hospitalization surgery. The former group received lidocaine/propofol/pethidine, while propofol/systemic opioid- and sevoflurane/propofol/systemic opioid-based anesthesia were administered to the latter group. Surgical stress response was evaluated based on time of operation and amount of bleeding during operation. Immune function was assessed based on natural killer (NK) cell activity, CD4/8 T cell ratio, and cytokine levels of IL-6 and IL-10 that were detected before surgery, after surgery, and on the first postoperative day.

**Results:**

Operation time did not differ between the two groups. Blood loss was significantly less for the hospitalization surgery group. No change in NK cell activity was observed for either group, although the CD4/8 T cell ratio increased transiently following day surgery. Levels of IL-6 increased significantly in both groups following surgery, and these levels tended to be higher in the hospitalization surgery group. One patient who underwent hospitalization surgery had higher levels of IL-10.

**Conclusions:**

There were few differences in immune response between the two groups, potentially since a majority of the hospitalization surgery patients received propofol-based anesthesia. We hypothesize that the use of volatile anesthetic/opioid analgesia in hospitalization surgery has a greater influence on immune function in breast cancer patients than local anesthetic/propofol-based anesthesia in day surgery.

## Background

Over the past decade, the relationship between anesthetic technique and cancer recurrence has been a major topic in oncologic surgery. The rationale for this discussion is based on the observation that volatile anesthetics and opioid analgesia suppress cell mediated immunity (CMI), thereby enabling residual or scattered tumor cells that remain after surgical resection to persist, grow, and metastasize to distant sites during the perioperative period [1–3]. A surgery-induced stress response also suppresses CMI, which further contributes to the proliferation of residual tumor cells or preexisting micrometastasis during surgery [4, 5]. In contrast, supplementation of regional anesthesia with general anesthesia (GA), or propofol-based anesthesia, reduces the surgical stress response and protects CMI, thereby reducing cancer recurrence [6]. Despite the fact that surgery/anesthetic technique-stimulated immunosuppression may be involved in cancer-related mortality, the mechanism by which anesthetic technique influences cancer outcome remains unproven.

Many retrospective studies, including meta-analyses, of the impact of anesthetic technique on cancer recurrence and cancer-related mortality have been conducted, and these have highlighted the potential benefit of using supplemental regional anesthesia or propofol as part of an anesthetic technique to reduce cancer recurrence following certain types of cancer surgery [7, 8]. In particular, a retrospective study showed that patient outcome in those receiving paravertebral or epidural anesthesia combined with GA was superior to that of those receiving GA/opioid analgesia [9]. Based on these data, a prospective randomized controlled trial (RCT) is currently underway to provide more definitive and conclusive evidence of anesthetic technique on cancer recurrence [10].

Day surgery is a common operative procedure for patients with breast cancer. In the United States and Europe, day surgery typically consists of partial resection of the breast (Bp) and sentinel lymph node biopsy (SNB). However, in Japan, hospitalization surgery is still common for cases of breast cancer. Here, immune responses associated with the anesthetic technique used for day surgery versus hospitalization surgery for breast cancer patients were prospectively analyzed. It was hypothesized that immune responses would differ according to anesthetic technique, and these responses would influence patient outcome.

## Patients and methods

### Patients

Between February 2012 and June 2012, 21 patients diagnosed with breast cancer underwent day surgery at the Hiroshima Mark Clinic. The surgeries included Bp/SNB (n = 17), Bp/axillar lymph node dissection (Ax) (n = 3), and total mastectomy of the breast (Bt)/Ax (n = 1). Between August 2012 and August 2014, 16 patients were diagnosed with breast cancer at the Hiroshima Mark Clinic and they preferred to undergo hospitalization surgery in Hiroshima City Hospital or Hiroshima University Hospital. These surgeries included Bp/SNB (n = 11), Bp/SNB/Ax (n = 3), and Bt/SNB (n = 2) (Table 1). Therefore, this prospective study examined day surgery versus hospitalization surgery according to patient preference, and the patients were not randomized. Different anesthetic techniques were performed for day surgery versus hospitalization surgery. To compare anesthesia-induced immune suppression and surgical stress response in the two groups, blood samples were collected from each patient to assess immune function. The number of patients for hospitalization surgery was low because the patients preferred day surgery rather than hospitalization surgery during the research period.

**Table 1:** Characteristics of the patient groups

Patient characteristics	Day surgery (n = 21)	Hospitalization surgery (n = 16)	P value
Age, y (median)	46 (35–71)	48.5 (38–75)	0.416*
Cancer stage			
I	10	14	
II	10	2	
III	1	0	
Neoadjuvant chemotherapy	4	1	
Surgical procedure			
Bp/SNB	17	11	0.041^+^
Bp/SNB/Ax	0	3	
Bp/Ax	3	0	
Bt/SNB	0	2	
Bt/Ax	1	0	
Subtype			
Luminal-A	9	5	0.763^+^
Luminal-B	9	7	
Luminal-HER2	3	1	
DCIS	0	3	
Anesthetic technique			
Lidocaine/propofol/pethidine	21		
Propofol TCI: fentanyl/remifentanil		7	
Propofol TCI: fentanyl		4	
Sevoflurane/propofol/fentanyl/remifentanil		4	
Sevoflurane/propofol/fentanyl		1	

*Ax* axillary lymph node dissection, *Bp* partial resection of the breast, *Bt* total mastectomy of the breast, *DCIS* ductal carcinoma in situ, *HER2* human epidermal growth factor-2, *SNB* sentinel lymph node biopsy, *TCI* target controlled infusion

* Mann–Whitney U test

^+^Chi square test

### Anesthetic technique

In the day surgery group, all 21 patients received local anesthetic/propofol-based anesthesia which consisted of lidocaine anesthetic, propofol anesthesia, and pethidine analgesia. Briefly, anesthesia was induced with 1 mg/kg propofol and 35 mg pethidine and was maintained with a continuous infusion of propofol at 6–8 mg/kg/h. Local anesthetic, 50–100 ml of 0.5% lidocaine, was used for local anesthesia. Since no tracheal intubation with muscle relaxant was performed, the patients recovered quickly. After resting in bed approximately 3–4 h after surgery, the patients returned home the same day. For the patients that underwent hospitalization surgery, 16 received propofol/opioid-based anesthesia or volatile/opioid-based anesthesia. Selection of anesthetic technique was at the discretion of the anesthesiologist involved. Seven patients received total intravenous anesthesia (TIVA) with propofol using target controlled infusion (TCI) of fentanyl/remifentanil, while propofol (3.0 μg/ml) and fentanyl (1–2 μg/kg) were administered at induction. Anesthesia was subsequently maintained with propofol (1.0–3.0 μg/ml) and remifentanil (0.25 μg/kg/min). Four patients who received TIVA with propofol using TCI of fentanyl were administered propofol (3.0 μg/ml) and fentanyl (1–2 μg/kg) at induction, and anesthesia was maintained with propofol (1.0–3.0 μg/ml). In four patients who received sevoflurane/propofol/fentanyl/remifentanil, propofol (2 mg/kg) and fentanyl (1–2 μg/kg) were administered at induction, and anesthesia was maintained with inhalation of sevoflurane (1.0–5.0%) and remifentanil (0.25 μg/kg/min). In one patient who received sevoflurane/propofol/fentanyl, propofol (3.0 μg/ml) and fentanyl (1–2 μg/kg) were administered at induction, and anesthesia was maintained with sevoflurane (1.0–5.0%). In the hospitalization surgery group, tracheal intubation was performed with the muscle relaxant, rocuronium (0.6 mg/kg), and the lungs were ventilated with a mixture of 1:2–3 O_2_/air. Postoperative analgesia was provided and it included non-steroidal anti-inflammatory drugs. The patients were treated for several days after surgery according to their needs.

### Immune function parameters

Blood samples were collected before and after surgery and 24 h postoperatively. Due to reasons attributed to patient preference and hospital situation, blood samples were not collected from all of the patients at each time point. However, all of the patients did provide informed consent for the collection of samples and their analysis. Immune function was evaluated based on natural killer (NK) cell activity, CD4/8 T cell ratio, and levels of cytokines IL-6 and IL-10 that were measured in these samples by SRL Inc. (Tokyo, Japan). In brief, lymphocyte subsets, including NK cells and CD4 and CD8 T cells, were analyzed by flow cytometry and plasma levels of IL-6 and IL-10 were measured with enzyme-linked immunosorbent assays.

### Statistical analysis

GraphPad InStat 3 (GraphPad, San Diego, CA, USA) was used for the statistical analyses performed. Continuous variables were analyzed by using the Mann–Whitney U test and discrete variables were analyzed by using the Chi squared test. The Mann–Whitney U test was also used to analyze differences between the groups. The results are presented as medians with quartiles. Differences in the median values of the paired sets were calculated with the Wilcoxon matched pairs test. *P* values less than 0.05 were considered statistically significant.

## Results

### Difference in patient background

Differences between the patients in the day surgery group and the hospitalization surgery group are summarized in Table 1. The day surgery group included a greater number of advanced clinical stage cases than the hospitalization surgery group. There was also a greater number of patients who underwent Bp/SNB in the day surgery group compared with the hospitalization surgery group. Four patients in the day surgery group and one patient in the hospitalization surgery group received neoadjuvant chemotherapy. There were three cases of ductal carcinoma in situ in the hospitalization surgery group.

### Surgery-induced stress response

**Fig. 1:**
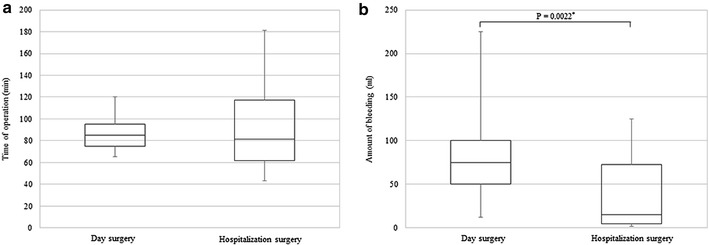
Comparison of operation time (**a**) and amount of bleeding (**b**) for patients undergoing day surgery versus hospitalization surgery for breast cancer. Median values are expressed with interquartile ranges in the box plot. *P < 0.05 versus hospitalization surgery (Mann–Whitney U test)

Surgery-induced stress responses were evaluated based on operation time and amount of bleeding during surgery. The median operation times for the day surgery group versus the hospitalization surgery group were 85.0 ± 10.0 and 81.5 ± 27.5 min, respectively, and they did not statistically differ. In contrast, the median amount of bleeding for the hospitalization surgery group was significantly less than that for the day surgery group (15.0 ± 33.7 ml vs. 75.0 ± 25.0 ml, respectively) (Fig. 1).

### Changes in immune function parameters

**Fig. 2:**
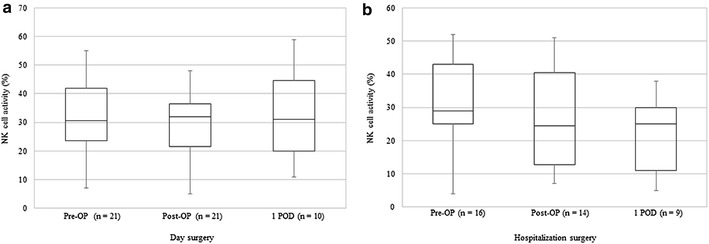
Changes in NK cell activity before surgery, after surgery, and on the first postoperative day in patients undergoing day surgery (**a**) versus hospitalization surgery (**b**) for breast cancer. Median values are expressed with interquartile ranges in the box plot. *Pre-OP* before operation, *Post-OP* after operation, *1 POD* first postoperative day

**Fig. 3:**
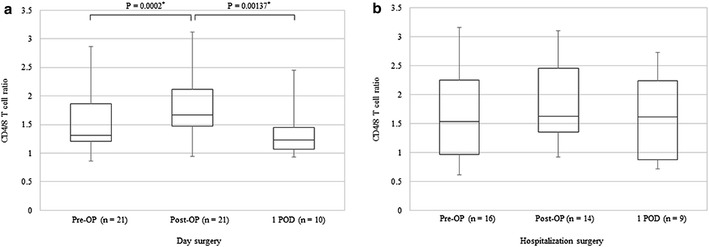
Changes in CD4/8 T cell ratio before surgery, after surgery, and on the first postoperative day in patients undergoing day surgery (**a**) versus hospitalization surgery (**b**) for breast cancer. Median values are expressed with interquartile ranges in the box plot. *P < 0.05 versus after surgery and on the first postoperative day (Wilcoxon matched pairs test). *Pre-OP* before operation, *Post-OP* after operation, *1 POD* first postoperative day

**Fig. 4:**
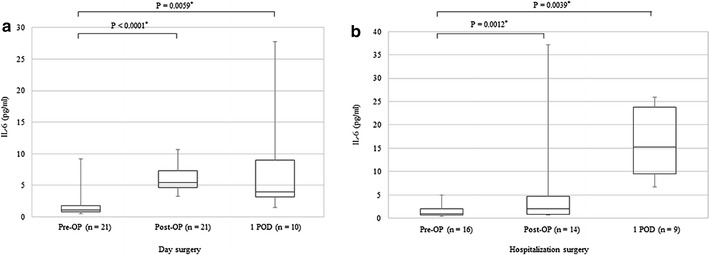
Changes in IL-6 levels before surgery, after surgery, and on the first postoperative day in patients undergoing day surgery (**a**) versus hospitalization surgery (**b**) for breast cancer. Median values are expressed with interquartile ranges in the box plot. *P < 0.05 versus after surgery and on the first postoperative day (Wilcoxon matched pairs test). *Pre-OP* before operation, *Post-OP* after operation, *1 POD* first postoperative day

Changes in immune function were evaluated based on measurements of NK cell activity, CD4/8 T cell ratios, and levels of cytokines, IL-6 and IL-10. Changes in the median values of NK cell activity before surgery, after surgery, and on the first postoperative day for the day surgery and hospitalization surgery groups were 30.5 ± 9.2 and 29.0 ± 9.0, 32.0 ± 7.5 and 24.5 ± 13.8, and 31.0 ± 12.3 and 25.0 ± 9.5%, respectively in each case. There were no statistically significant differences between the groups at any of the time points assayed. However, when the median values of NK cell activity at baseline before surgery were compared for the two groups, the values for the hospitalization surgery group tended to decline after surgery while the values for the day surgery group remained largely unchanged (Fig. 2). The mean CD4/8 T cell ratios at the three sampled time points were 1.31 ± 0.32 and 1.53 ± 0.64, 1.67 ± 0.34 and 1.63 ± 0.55, and 1.23 ± 0.16 and 1.61 ± 0.68 for the two groups, respectively in each case. These data demonstrate that the mean CD4/8 T cell ratios for the day surgery group transiently increased and then declined below baseline level on the first postoperative day (Fig. 3). In contrast, the median CD4/8 T cell ratios in the hospitalization surgery group did not significantly differ after surgery or on the first postoperative day compared with the values before surgery. The median IL-6 levels for the day surgery and hospitalization surgery groups were 1.1 ± 0.5 and 1.0 ± 0.65, 5.4 ± 1.35 and 2.0 ± 1.94, and 3.9 ± 2.95 and 15.3 ± 7.15 pg/ml, respectively in each case. Thus, the IL-6 levels significantly increased from baseline levels after surgery and on the first postoperative day in both groups (Fig. 4), while the median IL-6 level after surgery tended to be higher in the hospitalization surgery group compared with the day surgery group. Regarding levels of the immunosuppressive cytokine, IL-10, they increased after surgery in one patient in the hospitalization surgery group who received propofol with TCI of fentanyl. In contrast, none of the patients in the day surgery group showed increases in IL-10 levels.

## Discussion

This study was conducted to evaluate whether the immune response in breast cancer patients that underwent day surgery versus hospitalization surgery was affected by the anesthetic technique used during surgery. Unexpectedly, the difference in immune response between the two groups that was assessed based on several parameters of immune function was small. One possible reason for this result is that the anesthetic technique used for the day surgery group included the administration of local anesthetic/propofol/pethidine, and the major anesthetic technique in the hospitalization surgery group was also propofol-based anesthesia in combination with opioid analgesia. However, there were some patients that received volatile anesthesia with opioid analgesia. Propofol has been shown to exert a protective effect on CMI, while volatile anesthetics suppress CMI [11]. Thus, the anesthetic technique used between day surgery and hospitalization surgery may have less influence on immune function following surgery in patients with breast cancer. Nevertheless, the day surgery patients exhibited a transient upregulation in their CD4/8 T cell ratios, no change in NK cell activity, a transient upregulation of IL-6 levels, and no increase in IL-10 levels. Thus, the anesthetics administered in the day surgery group produced less of an effect on the patients’ immune response compared with the anesthetics administered for the hospitalization surgery group, even though a greater number of advanced cancer stage cases were included in the day surgery group.

Surgery has been shown to suppress CMI in proportion to the degree of surgical manipulation and intensity of the stress response involved. In particular, stimulation of the hypothalamic–pituitary–adrenal (HPA)-axis and the sympathetic nervous system leads to reduced NK cell activity and T cell responses [12]. In response to surgical stress, catecholamines and prostaglandins are released. The downstream effects of this release include a decrease in immunostimulating cytokines such as IL-2, IL-12, and interferon-γ, and an increased production of the anti-inflammatory cytokine, IL-10 [4, 13]. IL-6 has a bifunctional role in tumor microenvironments by exerting a protumor response with respect to tumor angiogenesis and an anti-tumor T cell response with respect to tumor growth [14]. In the present study, surgical stress response was evaluated based on operation time and amount of bleeding. The operation times did not significantly differ between the two groups, although greater bleeding occurred in the day surgery group compared with the hospitalization surgery group. The latter observation may represent a greater surgical stress response compared with the hospitalization surgery group. Another potential reason for the difference in bleeding may be, in part, due to differences in the stage and extent of surgery that were involved with each of the two groups. For example, the day surgery group included a greater number of more advanced cancer stage cases than the hospitalization group. Nevertheless, the immune responses in the day surgery group (based on cytokine levels and CD4/8 T cell ratios) exhibited less change compared to the hospitalization surgery group, thereby suggesting that day surgery may involve less immunosuppression than hospitalization surgery.

Propofol has been shown to mediate anti-inflammatory and anti-tumor effects on tumor progression, while volatile anesthetics have exhibited a protumor effect [2, 15]. In fact, propofol has been shown to suppress NK cell activity and increase the activity of cytotoxic T-lymphocytes, while decreasing the secretion of pro-inflammatory cytokines and inhibiting the function of cyclooxygenase-2 and prostaglandin E_2_ [16, 17]. In contrast, propofol has not been found to affect helper T1/helper T2 ratios and it appears to preserve IL-2/IL-4 and CD4/8 T cell ratios, thereby attenuating an adverse immune response induced by surgery [18]. Meanwhile, sevoflurane has been shown to induce apoptosis in T-lymphocytes and increase the activity of hypoxia inducible factor 1-α in experimental models [19, 20]. Patients who received sevoflurane/opioid anesthesia also exhibited increased levels of protumorigenic factors, such as IL-1β and matrix metalloproteinases, compared with patients who received propofol/paravertebral anesthesia for breast cancer surgery [21]. Taken together, these findings suggest that propofol-based anesthesia may have less of an effect on immunosuppression and tumor development compared with volatile anesthesia in breast cancer surgery. To investigate a potential association between propofol anesthesia and cancer recurrence, a recent study compared the administration of opioid analgesia during the perioperative period with the administration of propofol for breast cancer patients undergoing mastectomy [22]. There were fewer cases of cancer recurrence in the latter compared with the sevoflurane group [22]. These results suggest that propofol anesthesia may reduce the risk of cancer recurrence during the first 5 years following mastectomy. Accordingly, a prospective RCT trial was designed and initiated as a multi-center study in November 2013 for patients undergoing radical surgery for breast cancer (NCT02089178). This study is ongoing and it is anticipated that the 1- and 5-year survival rates will indicate the impact of anesthetic technique on cancer recurrence following breast cancer surgery.

Opioids are an important analgesia during surgery and also suppress CMI. In experimental models, fentanyl and remifentanil have been found to decrease NK cell activity [23, 24]. Similarly, morphine has been shown to suppress NK cell activity, while also inhibiting T cell differentiation and promoting apoptosis in lymphocytes [25]. However, differences in immunosuppression with the use of synthetic opioids have been observed. It is predicted that pethidine may mediate less of an immunosuppressive effect compared with other opioids, particularly compared with a combination of fentanyl and remifentanil or morphine alone [26].

Local anesthetics have been found to suppress the proliferation of several types of cancer cells, potentially by blocking voltage-gated sodium channels which are highly expressed in a variety of cancer cells, including breast cancer cells [27]. When lidocaine was administered at clinically relevant concentrations both in vitro and in vivo [28], apoptosis in breast tumor cells was induced, thereby suggesting a beneficial action of local anesthetics on cancer cells in breast cancer surgery. In another study, lidocaine was found to demethylate the DNA of breast cancer cell lines that were positive or negative for expression of estrogen receptor to mediate a tumor-suppressive effect [29]. It is hypothesized that infiltrative anesthetics have membrane-stabilizing activity, as observed with lidocaine, and that they effectively inhibit the invasive ability of human cancer cells at concentrations used in surgical operations [30]. When cancer cells were treated in vitro with lidocaine at clinically relevant concentrations, NK cell cytotoxicity was augmented via the release of lytic granules that contained perforin and granzyme B [31]. These results suggest that local administration of lidocaine may be beneficial for breast cancer surgery by enhancing NK cell cytotoxicity. Moreover, the resulting cytotoxic effect on cancer cells could lead to an inhibition of metastatic potential in tumor progression.

## Conclusions

Currently in Japan, most surgeries for breast cancer involve hospitalization. However, we wanted to investigate whether the use of local anesthetics in combination with anesthetic sedation can result in decreased cancer recurrence. Therefore, immune function was evaluated in this preliminary and prospectively designed study that included a small sample of breast cancer patients who received local anesthetic/anesthetic sedation for day surgery versus GA for hospitalization surgery. The limitations of this study included the small sample of patients that were examined, the lack of uniformity in the type of anesthetic technique that was applied to the patients undergoing hospitalization surgery, and only a few patients received volatile anesthesia. Moreover, the present study was not designed according to anesthesia type. However, the results of this study provide preliminary evidence that local anesthetic/propofol-based anesthesia that is used in day surgery has less of an influence on immune function in breast cancer patients compared with volatile anesthetic/opioid analgesia that is used in hospitalization surgery. In addition, the present results support our current efforts to design a RCT study that will verify whether the use of local anesthetic/anesthetic sedation without opioids and GA, including TIVA or volatile anesthetics with opioids, affects immune function and cancer recurrence rate in breast cancer patients undergoing breast conserving surgery.

## Abbreviations

Ax: axillar lymph node dissection
Bp: partial resection of the breast
Bt: total mastectomy of the breast
CMI: cell mediated immunity
GA: general anesthesia
HPA: hypothalamic–pituitary–adrenal
NK: natural killer
RCT: randomized controlled trial
SNB: sentinel lymph node biopsy
TCI: target controlled infusion
TIVA: total intravenous anesthesia
